# Supratentorial Lymphocytic Inflammation with Parenchymal Perivascular Enhancement Responsive to Steroids (SLIPPERS)—Does it Really Exist?

**DOI:** 10.3390/brainsci13081191

**Published:** 2023-08-11

**Authors:** Fernando Freua, João Vitor Mahler, Pedro Lucas Grangeiro de Sá Barreto Lima, Iuri Santana Neville, Leonardo Barreira Portella, Victor Hugo Rocha Marussi, Carmen Lucia Penteado Lancellotti, Paulo Ribeiro Nobrega, Guilherme Diogo Silva

**Affiliations:** 1 Beneficência Portuguesa Hospital, São Paulo 01323-001, Brazil; fernando.freua@hc.fm.usp.br (F.F.); iuri.neville@hc.fm.usp.br (I.S.N.); l.leonardo.barreira@gmail.com (L.B.P.); vhmarussi@hotmail.com (V.H.R.M.); 2 Clinics Hospital, Faculty of Medicine, University of Sao Paulo, São Paulo 05403-010, Brazil; joao.mahler@fm.usp.br (J.V.M.); guilherme.diogo@hc.fm.usp.br (G.D.S.); 3 Faculty of Medicine, Federal University of Ceara, Fortaleza 60430-160, Brazil; pedro.lucas@alu.ufc.br; 4 Faculty of Medical Sciences, Santa Casa de São Paulo, São Paulo 01224-001, Brazil; luciapl@icloud.com; 5 Division of Neurology, Walter Cantidio University Hospital, Federal University of Ceara, Prof. Costa Mendes St., 1608, 4th Floor, Rodolfo Teófilo, Fortaleza 60430-140, Brazil; 6 Faculty of Medicine, Centro Universitário Christus, Fortaleza 60160-230, Brazil

**Keywords:** SLIPPERS, CLIPPERS, supratentorial CLIPPERS, glial fibrillary acid protein, neuroimaging, neuroinflammatory disease

## Abstract

Supratentorial Lymphocytic Inflammation with Parenchymal Perivascular Enhancement Responsive to Steroids (SLIPPERS) is a rare variant of the CLIPPERS spectrum with less than ten reports published so far. There is ongoing discussion regarding whether SLIPPERS is a disease entity on its own or just an acronym encompassing many underlying diagnoses, such as sarcoidosis, vasculitis and anti-glial fibrillary acidic protein (GFAP)-associated disease. A 40-year-old woman presented with episodes of language and attention impairment. Magnetic resonance imaging (MRI) revealed T2/FLAIR hyperintense lesions in the subcortical white matter associated with a micronodular, curvilinear perivascular contrast-enhancement. Alternative diagnoses were excluded. There was a remarkable response to steroids. A relapse occurred after six years, and the biopsy showed perivascular T-cell lymphocytic infiltrate, without granulomas, vasculitis, or neoplasia. There was complete resolution of the relapse after steroids. This case represents the longest reported follow-up of a patient diagnosed with SLIPPERS, and brain biopsy after 6 years did not suggest alternative diagnoses. This report contributes to the discussion regarding the possibility that exclusive supratentorial CLIPPERS-like pathology might be an isolated disease entity, but more biopsy-proven cases with a longer follow-up are needed to support this hypothesis. Recently, GFAP astrocytopathy has been characterized and might correspond to a significant number of cases previously diagnosed as CLIPPERS or SLIPPERS.

## 1. Introduction

Chronic Lymphocytic Inflammation with Pontine Perivascular Enhancement Responsive to Steroids (CLIPPERS) presents as idiopathic steroid-responsive T-CD4 lymphocyte infiltrates associated with pontine perivascular gadolinium-enhancing punctate lesions [1]. Few case reports and series have been published on this disease, limiting our knowledge of the full clinical spectrum and pathophysiology of this entity [2]. In 2015, exclusive supratentorial lesions with radiological and pathological features similar to CLIPPERS were described and received the acronym Supratentorial Lymphocytic Inflammation with Parenchymal Perivascular Enhancement Responsive to Steroids (SLIPPERS) [3]. Since then, less than ten case reports have been described. Little is known about the long-term outcomes and prognosis of SLIPPERS and there is ongoing discussion whether it constitutes a disease entity on its own or just an acronym encompassing many underlying diagnoses, such as sarcoidosis, central nervous system (CNS) vasculitis and possibly anti-GFAP associated disease.

Here we report clinical, radiological, and pathological features of a well-documented case fulfilling the proposed criteria for SLIPPERS with a six-year follow-up. In this case, the biopsy was compatible with previous findings in SLIPPERS and excluded common differential diagnoses like CNS vasculitis and granulomatous diseases.

## 2. Methods

This is a retrospective report of a patient evaluated in a specialized neuroimmunology center in Brazil. Informed consent for publication was obtained for the patient. All ancillary tests were performed for the regular follow-up at the discretion of the attending physician. Brain MRI was evaluated by a neuroradiologist. Pathological specimens were evaluated by experienced neuropathologists.

## 3. Case Report

A 34-year-old woman with a past medical history of vitiligo presented with episodes of speech/language impairment for the last six months. Family history was significant for a sister diagnosed with Sydenham chorea. Neurological examination demonstrated only brisk reflexes on the left-side.

**Figure 1:**
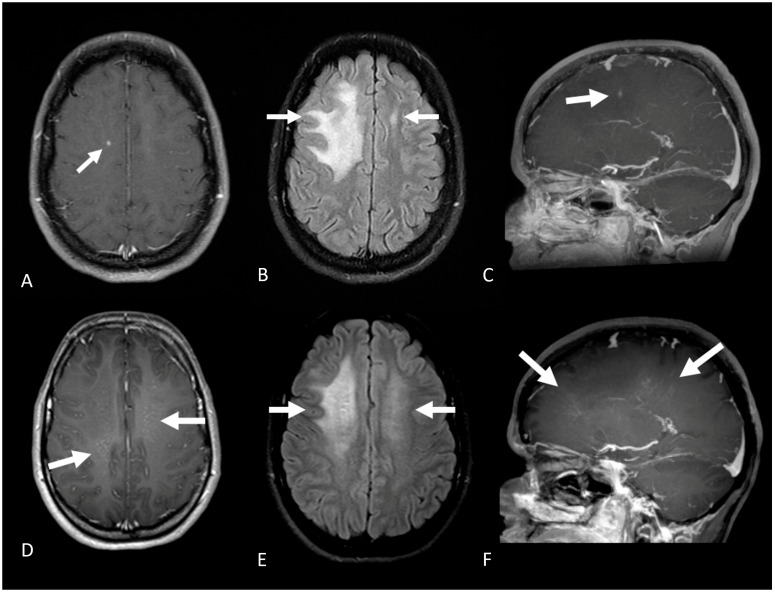
Axial (**A**) T1 post-contrast brain MRI, (**B**) axial FLAIR and (**C**) sagittal post-contrast T1 weighted MRI show a punctate enhancing lesion in the right superior frontal gyrus with adjacent vasogenic edema (white arrows). After treatment, the small punctate lesion disappeared (**D**) with reduction of the vasogenic edema (**E**); however, multiple linear enhancing lesions with perivascular distribution were seen in the deep white matter (white arrows in (**F**)).

Magnetic resonance imaging showed bilateral T2/FLAIR hyperintense lesions in the frontal subcortical white matter associated with a micronodular, curvilinear perivascular pattern of contrast-enhancement (Figure 1). Cerebrospinal fluid (CSF) demonstrated a lymphocytic pleocytosis of 9 cells/mm^3^, and normal protein and glucose levels. Protein electrophoresis demonstrated an increase in CSF IgG levels. Oligoclonal bands were absent. Chest and abdominal computed tomography scans revealed no abnormalities. Serum ANA, Rheumatoid Factor, Anti-Ro, Anti-La, Anti-RNP, anti-dsDNA, Anti-Sm, ANCA, ASCA, C3, C4, ACE, AgHBs, Anti-HCV, HIV serology, HTLV serology, and FTA-abs were normal.

The patient received 1 g/day of methylprednisolone for 5 days, considering a possible diagnosis of SLIPPERS and the absence of infection. The administration of high-dose steroids led to complete clinical and radiological improvement after 3 months (Figure 1). We did not start any long-term immunotherapy due to the predominantly monophasic course of this disease. Semestral follow-up did not reveal further symptoms.

Two years after disease onset, the patient became pregnant. Pregnancy and delivery occurred without maternal or neonatal complications. No neurological symptoms occurred during this period, as well as during the puerperium.

**Figure 2:**
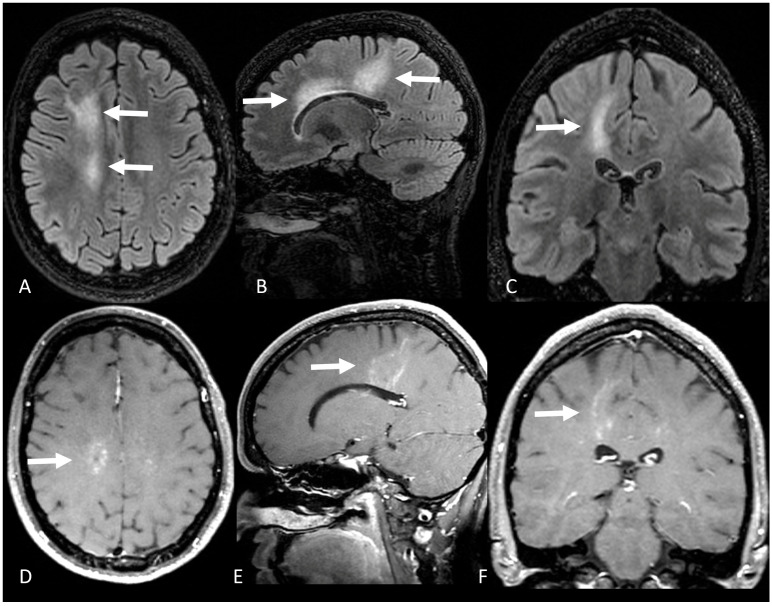
(**A**) Axial, (**B**) sagittal, and (**C**) coronal FLAIR shows hyperintensity in the right fronto-parietal subcortical and periventricular white matter (white arrows). (**D**) Axial, (**E**) sagittal, and (**F**) coronal T1-weighted gadolinium-enhanced images shows patchy nodular and curvilinear pattern of contrast enhancement in the right fronto-parietal subcortical and periventricular white matter (white arrows).

**Figure 3:**
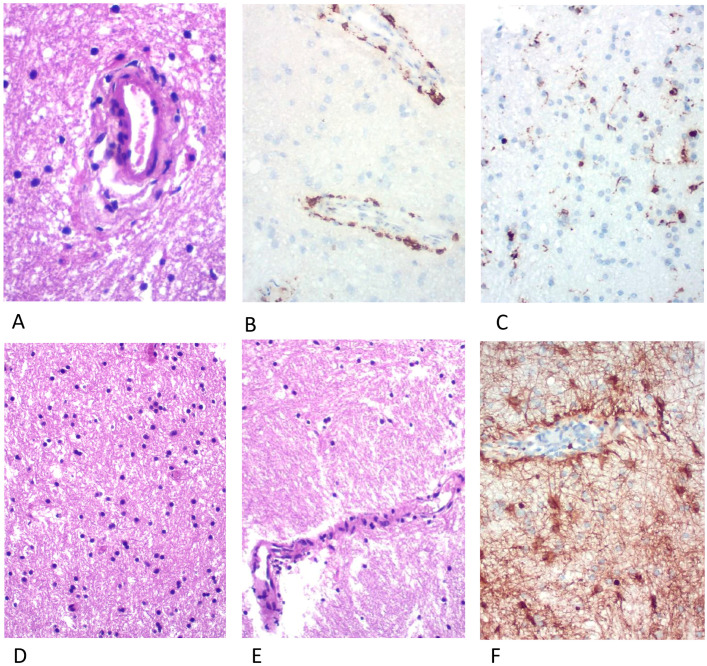
(**A**) Discrete inflammatory process in the cerebral white matter with perivascular CD3+ lymphocyte infiltration (**B**), frequent CD68+ macrophages (**C**), vasogenic edema, focal myelin pallor (**D**,**E**) and GFAP+ staining showing reactive astrocytes (**F**). No granulomatous or neoplastic processes were seen.

Six years after the disease onset, she presented with cognitive impairment, particularly in attention, associated with headache. Magnetic resonance imaging revealed new areas of nodular, perivascular T2/FLAIR hyperintensities in the frontal subcortical and deep white matter with areas of nodular and linear contrast enhancement (Figure 2). Considering a disease relapse, we performed a brain biopsy. Histopathology demonstrated perivascular T-cell predominant lymphocytic infiltrate, without evidence of granulomas, vasculitis, or neoplastic processes (Figure 3). A new course of methylprednisolone 1 g/day for 3 days led to complete symptom improvement. Long-term immunotherapy was not started at that moment due to the unavailability of clear guidelines for this condition. Close follow-up was maintained and she had no further relapses for 2 years.

## 4. Discussion

This case represents the longest reported follow-up of a patient diagnosed with SLIPPERS, and a brain biopsy after 6 years of disease onset did not suggest any alternative diagnoses. This report contributes to the discussion regarding the possibility that exclusive supratentorial CLIPPERS-like pathology might be an isolated disease entity, but more biopsy-proven cases a with longer follow-up are needed to support this hypothesis.

We also discuss characteristic neuroimaging findings, possible differential diagnoses and the possibility of relapses in this pathology. Of note, this was the first report of a successful pregnancy in a patient diagnosed with SLIPPERS, and they did not show worsening or relapse during pregnancy or puerperium.

In the original CLIPPERS series, all patients’ MRIs exhibited a distinct pattern of punctate and curvilinear enhancement peppering the pons and extending variably into the medulla, brachium pontis, and mid-brain. In some cases, a milder radial pattern of identical enhancing lesions extended into the basal ganglia and inferior cerebellar white matter, corpus callosum, and spinal cord. Lesions became fewer and smaller as distance from the pons increased [1]. However, CLIPPERS-like pathology and imaging patterns were identified in the spinal cord, midbrain, cerebellum, medulla and in supratentorial regions [3,4,5,6]. Additionally, multiple extrapontine inflammatory changes that were not seen with conventional 3-Tesla imaging were detected with 7-Tesla magnetic resonance imaging in four CLIPPERS patients, emphasizing that subclinical pathology may occur beyond the pons [7].

Diagnostic criteria proposed for CLIPPERS require enhancing lesions predominating in the pons [8]. However, some cases with exclusive supratentorial involvement and similar neuropathological findings have been reported since then [3,9,10,11,12]. Hence, the term SLIPPERS has been proposed to characterize patients with similar neuroimaging and pathological findings involving predominantly the supratentorial compartment [3].

SLIPPERS is very rare and to the best of our knowledge, only six cases of exclusive supratentorial lesions have been reported (Table 1). The appearance of an alternative diagnosis during the follow-up for CLIPPERS is high: after a median follow-up of 50 months, 13 out of 42 cases initially diagnosed with CLIPPERS received another diagnosis such as lymphoma or autoimmune gliopathies [8]. It is reasonable to believe the same occurs with SLIPPERS. A systematic review of 140 cases of CLIPPERS demonstrated that 16% of patients were eventually diagnosed with cancer, particularly hematological neoplasia [13]. Nevertheless, we excluded most autoimmune and neoplastic conditions in this case with a brain biopsy performed after 6 years of follow-up, leading us to believe that this patient would still fall in the SLIPPERS diagnostic criteria. However, as the pathophysiological mechanisms of CLIPPERS and SLIPPERS are still not fully understood, it is possible that other still-unknown disease processes are responsible for this patient’s findings.

Since its first description in 2016, autoimmune GFAPastrocytopathy has been increasingly recognized as a cause of autoimmune meningoencephalitis with particular neuroimaging and pathologic findings [14]. Autoimmune GFAP astrocytopathy may mimic CLIPPERS both clinically and radiologically. Cases of CLIPPERS reported prior to 2016 may have been caused by an autoimmune GFAP astrocytopathy and the availability of the antibody test currently allows for the distinction between these two uncommon disorders.

CLIPPERS neuropathology demonstrates extensive lymphocytic inflammation (T cells predominance) with perivascular predominance and parenchymal diffuse infiltration, associated with an absence of myelin loss or focal secondary myelin loss [8]; there is no definite etiology and pathophysiology for these changes. Similarly, some cases of GFAP astrocytopathy neuropathology showed extensive inflammation (infiltration of lymphocytes, monocytes, and neutrophils) around the vessels [15], which was consistent with linear perivascular radial gadolinium enhancement characteristics, but astrocyte loss, neuron loss, and demyelination were not always observed. However, unlike CLIPPERS, B cells and plasma cells were found in the lesion region Virchow–Robin spaces in some GFAP astrocytopathy specimens. The present case had no B-cell infiltrates on biopsy. Unfortunately, anti-GFAP antibody analysis could not be performed due to accessibility issues.

Even in patients diagnosed with CLIPPERS/SLIPPERS, long-term follow-up is required for necessary diagnostic revision at each new attack and the GFAP antibody test is advised for differential diagnosis, particularly when the patient presents with linear perivascular radial gadolinium enhancement.

Little is known about the long-term outcomes of SLIPPERS. However, our case demonstrated that patients could present relapses several years after disease onset. Moreover, almost 60% of patients with CLIPPERS show relapses [13]. Although this data may prompt a discussion about the need for chronic immunotherapy, our patient presented an excellent response to high-dose steroids in both events, suggesting a favorable overall long-term prognosis even with relapses.

Pregnancy can ameliorate autoimmune diseases such as rheumatoid arthritis but can also exacerbate or have no effect on other conditions such as lupus erythematosus [16]. The disease activity of CLIPPERS and SLIPPERS during pregnancy and post-partum is unknown. Our patient had an uneventful pregnancy, but these findings need to be evaluated in future case series.

Our report has limitations that must be addressed. First, the comprehension of the pathophysiology is limited and the concept that SLIPPERS represents part of the clinical spectrum of CLIPPERS is based on radiological and pathological findings. Also, multiple autoimmune and neoplastic disorders can present the radiological and pathological findings of SLIPPERS, reinforcing the importance of long-term follow-up in case reports of the disease to evaluate alternative diagnoses.

Recently, GFAP astrocytopathy has been better characterized and might correspond to a significant number of cases previously diagnosed as CLIPPERS or SLIPPERS. Unfortunately, we were not able to test for anti-GFAP antibodies due to unavailability of antibody testing at the initial presentation and difficulties in access to antibody research during relapse. This is an important limitation as clinical and radiological aspects of this case may overlap with anti-GFAP and biopsy findings in GFAP astrocytopathy have not been extensively documented. Furthermore, a single case report has limited generalizability and lacks predictive value for disease progression estimates.

## 5. Conclusions

We reported the longest follow-up in a biopsy-compatible SLIPPERS case. This case highlights the necessity to investigate and exclude potential alternative diagnosis, particularly CNS vasculitis, hematologic malignancies and granulomatous diseases, in an expanding list of differential diagnoses. Recently described GFAP astrocytopathy may correspond to a significant number of cases previously diagnosed as CLIPPERS or SLIPPERS, and testing for GFAP-antibodies is advised even in long-term or relapsing disease. Case series with a longer follow-up and testing for anti-GFAP antibodies could help us understand if SLIPPERS is a disease entity on its own and provide guidance for treatment and follow-up of these cases.

## Figures and Tables

**Table 1:** Case reports of exclusive Supratentorial Lymphocytic Inflammation with Parenchymal Perivascular Enhancement Responsive to Steroids (SLIPPERS).

	(Armand et al., 2015 [3]) (*n* = 2)	(Horng et al., 2017 [9]) (*n* = 1)	(Sudhakar et al., 2021 [10]) (*n* = 1)	(Picarelli et al., 2021 [11]) (*n* = 1)	(Vattoth et al., 2022 [12]) (*n* = 1)
Age	N/A ^1^	56	71	39	21
Sex	N/A ^1^	Male	Male	Female	Male
Clinical presentation	Seizures, headache and hemiparesis	Cognitive impairment	Cognitive impairment and hemiparesis	Headache and seizures	Seizure, dizziness and left homonymous hemianopia
Location	N/A ^1^	Bilateral PVWM lesions, including the amygdala and hippocampus	Right occipital lobe, right precentral gyrus, and multiple small lesions in PVWM	Subcortical and PVWM lesions in the right frontoparietal and insular lobes	Right parieto-occipital subcortical and PVWM, extending to the splenium
Follow up of more than 2 years	No	No	No	No	No

^1^ N/A Not available.

## Data Availability

All data were included in the manuscript.
